# Dietary associations with reduced epigenetic age: a secondary data analysis of the methylation diet and lifestyle study

**DOI:** 10.18632/aging.206240

**Published:** 2025-04-17

**Authors:** Jamie L. Villanueva, Alexandra Adorno Vita, Heather Zwickey, Kara Fitzgerald, Romilly Hodges, Benjamin Zimmerman, Ryan Bradley

**Affiliations:** 1 School of Nursing, University of Washington, Seattle, WA 98195, USA; 2 Helfgott Research Institute, National University of Natural Medicine, Portland, OR 97201, USA; 3 Colorado State University, Department of Food Science and Human Nutrition, Fort Collins, CO 80526, USA; 4 Institute for Functional Medicine, WA 98003, USA; 5 College of Nutrition, Sonoran University, Tempe, AZ 85282, USA; 6 Herbert Wertheim School of Public Health and Human Longevity Sciences, University of California, San Diego, CA 92093, USA

**Keywords:** aging, epigenetics, DNA methylation, diet, biological clock

## Abstract

Background: Aging is the primary risk factor for developing non-communicable chronic diseases, necessitating interventions targeting the aging process. Outcome measures of biological aging used in these interventions are mathematical algorithms applied to DNA methylation patterns, known as epigenetic clocks. The Methylation Diet and Lifestyle study was a pilot randomized controlled trial of a diet and lifestyle intervention that utilized epigenetic age as its primary outcome, measured using Horvath’s clock. Significant reductions in epigenetic age post-intervention were observed but with notable variability.

Purpose: This research aimed to identify dietary components associated with epigenetic age change across groups. Contributing factors to variability, such as weight changes and baseline differences in chronological and epigenetic age, were explored.

Results: In hierarchical linear regression, foods investigated as polyphenolic modulators of DNA methylation (green tea, oolong tea, turmeric, rosemary, garlic, berries) categorized in the original study as methyl adaptogens showed significant linear associations with epigenetic age change (B = -1.21, CI = [-2.80, -0.08]), after controlling for baseline epigenetic age acceleration and weight changes. Although the intervention group lost significantly more weight than the control group, these changes were not associated with epigenetic age changes in the regression model. These findings suggest that consuming foods categorized as methyl adaptogens may reduce markers of epigenetic aging.

## INTRODUCTION

Chronological age is the leading risk factor for developing noncommunicable chronic diseases (NCDs), including cardiovascular disease, diabetes, cancer, and neurodegenerative diseases [1]. In response to the increasing prevalence of chronic disease in a rapidly aging population, geroscience research aims to elucidate the biological mechanisms of aging and develop multi-system interventions to prevent or delay the onset of age-related chronic diseases. Alterations in cellular function regulating inflammation, cell cycle regulation, cellular energy production, detoxification, and remediation of oxidative stress are mechanistically connected to established chronic diseases of aging. The regulation of gene expression controls these processes, with epigenetic regulation potentially malleable [2, 3]. Consequently, epigenetics is a notable area of interest within geroscience [4].

Measures of epigenetic age, determined by analyzing DNA methylation patterns using mathematical algorithms, have been proposed to be markers of biological aging. DNA methylation, the addition of methyl groups to DNA molecules at cytosine-5 CpG dinucleotides, is crucial in maintaining genomic integrity and changes predictably during aging [5]. First-generation measures of epigenetic age emerged a decade ago as researchers sought to predict chronological age using DNA methylation [6–8]. In 2013, Horvath created the first multi-tissue algorithm to measure biological aging and explored the associations between accelerated epigenetic age and disease in several cohorts, first introducing the term “epigenetic clock” into the scientific lexicon [8]. The results suggested that epigenetic age could be used as a marker of biological aging in research. In the subsequent years, second, and third-generation clocks were developed using DNA methylation to target biological markers of phenotypic aging, potentially demonstrating increased accuracy when predicting morbidity and mortality in epidemiological data [9–12].

Aging clocks have since been classified as intrinsic or extrinsic measures of epigenetic aging. Intrinsic epigenetic age (e.g., Horvath’s clock) characterizes innate aging, as first-generation measures were developed using machine learning techniques, like elastic net or lasso, to regress chronological age onto DNA methylation. Extrinsic epigenetic age algorithms (second and third-generation clocks) were developed by incorporating chronological age, immunological, metabolic aging-related changes (C-reactive protein, white blood cell count, serum glucose, etc.), and lifestyle factors (smoking history) into regression models.

The Methylation Diet and Lifestyle study was a randomized controlled trial in which changes in epigenetic age, measured using Horvath’s 2013 algorithm, were the primary outcome measure to determine the efficacy of an eight-week diet and lifestyle intervention in healthy middle-aged men [13]. Participants in the treatment group were randomized to a multi-modal intervention that included dietary and sleep recommendations, exercise, and daily meditation. The omnivorous but plant-centered diet regimen was formulated to include foods containing vitamins and polyphenolic compounds, shown in preclinical studies, that act as cofactors or substrates in DNA methylation pathways [14–18]. Participants were also instructed to avoid added sugar, trans-fats, grains, legumes, dairy, and alcohol. At the end of the trial, the intervention group was, on average, 2.04 years younger than their baseline epigenetic age (*p* = 0.043). The control group was, on average, 1.10 years older than their baseline measurements (*p* = 0.191), leading to a between-group difference of 3.14 years (*p* = 0.018) favoring the intervention [13].

Although the treatment group exhibited a significant reduction in epigenetic age at the end of the trial, a notable variability in epigenetic age changes was observed in both the treatment and control groups. Within the treatment group, the range of epigenetic age change was 17.36 years, with the most significant reduction in epigenetic age being -8.84 years, whereas the most significant increase in epigenetic age was +8.52 years. The control group had a range of 13.29 years, from a reduction of -6.61 years to an increase of +6.68 years.

The main objective of this research is to investigate diet as one potential contributor to the observed variability in epigenetic age changes, focusing on the association between participant-reported consumption of study-recommended and restricted foods and changes in epigenetic age using self-reported dietary data and methyltetrahydrofolate (MF) blood levels. Recent work evaluating the beneficial health effects of Mediterranean diet interventions compared to following healthy dietary guidelines on the epigenome highlighted folate levels as a marker of green vegetable intake and a mediator of one-carbon metabolism, a process critical to epigenetic regulation [13].

Secondarily, we aim to examine factors potentially influencing epigenetic age change that were not explored in the original study, including caloric restriction and baseline epigenetic age acceleration. Caloric restriction is a well-known method to reverse cellular aging [19]. Although the study diet did not limit caloric intake, those who strictly adhered to the plant-focused intervention may have unintentionally reduced their calories, potentially resulting in weight loss. As such, weight changes within the treatment and control groups require interrogation. Based on previously reported findings, baseline differences in epigenetic and chronological age will be assessed for association with epigenetic age changes [20]. In summary, this secondary data analysis explores dietary factors, weight confounders, and baseline differences between participant epigenetic age and chronological age concerning the existing evidence as predictors of intervention response.

## RESULTS

### Participant characteristics

Participants were 43 healthy adult men aged 50-72 from Portland, OR, USA; most were white (81%) and highly educated, with 41% reporting a graduate degree (Table 1). Six participants dropped out of the study: four from the intervention group (n = 18) and two from the control group (n = 20), leaving data from 38 individuals for analysis.

**Table 1:** Baseline participant characteristics.

**Baseline characteristics**	**Treatment group n = 21**		**Control group n = 22**	
	**Value**	**%**	**Value**	**%**
**Age (mean ± SD)**	58.2 ± 6.12		60.3 ± 6.68	
**Race**				
Black or African American			2	9.1
Asian or Asian American	3	13.6	1	4.5
Caucasian	18	81.8	18	81.8
More than one race			1	4.5
**Education Level**				
Highschool			3	0.14
Some university	1	0.05	1	0.05
2-year university	2	0.09	1	0.05
4-year university	6	0.27	4	0.18
Some graduate school	2	0.09	4	0.18
Graduate degree	11	0.50	9	0.41

### Consumption of dietary variables

**Figure 1:**
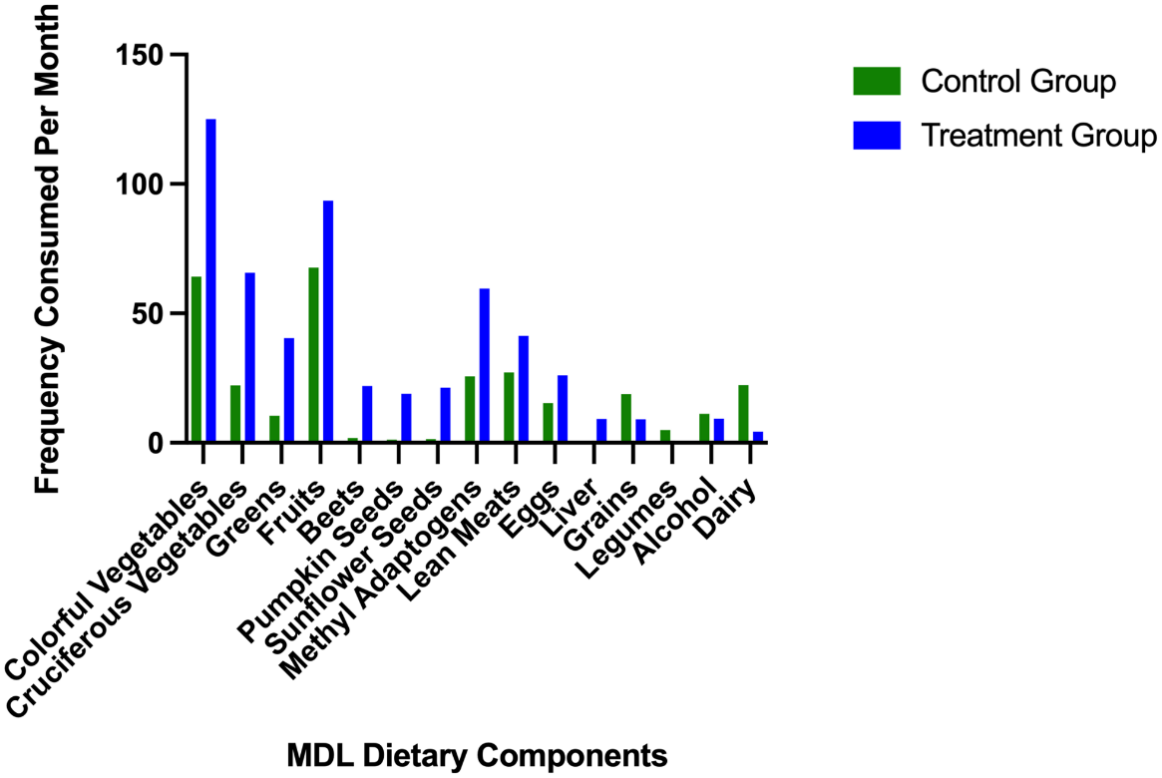
Average frequency of consumption of study recommended and restricted foods.

Participants in the intervention group were directed to eat various healthful plant foods and lean meats while avoiding alcohol, sugar, trans fats, and dairy (Table 2). Subjects in the control group maintained their regular eating pattern, leading to significant differences between consumption of dietary variables (Table 3). The mean consumption per month of recommended and restricted foods in the treatment and control groups is illustrated in Figure 1. Colorful vegetables and fruit were the most frequently consumed food groups. The treatment group consumed colorful vegetables an average of 125 times (*SD* = 60.16) and fruit an average of 93.5 times (*SD* = 50.53) during the last month of the study. While the control group consumed colorful vegetables an average of 65.23 (*SD* = 53.09) and fruit an average of 67.7 times (*SD* = 46.88) within the last month. As expected, the control group consumed little to none of certain study-specific recommended foods (i.e., liver, beets, pumpkin, and sunflower seeds) and more study-restricted foods (grains, dairy, alcohol, and legumes) than the treatment group. The most frequently consumed foods in the restricted category in the control group were dairy products, 22 times (*SD* = 15.87), and grains, 18.75 times (*SD* =15.03). The treatment group consumed grains and alcohol an average of ~9 times (*SD* = 12.76), dairy an average of 4.25 times (*SD* =8.20), and legumes less than once in the last month of the study (*SD* = 1.36).

**Table 2:** Recommendations for dietary intake in the methylation diet and lifestyle trial.

**Study dietary recommendations**		
**Dietary components/ variables**	**Serving size**	**Frequency**
Liver	3 oz	3x per week
Eggs	1 egg	5-10x per week
Dark Leafy Greens	2 cups	daily
Cruciferous Vegetables	2 cups	daily
Colorful Vegetables	3 cups	daily
Beets	1-2	daily
Pumpkin Seeds	4 tbsp (1/4 cup)	daily
Sunflower Seeds	4 tbsp (1/4 cup)	daily
Methyl Adaptogens:		1+ serving daily
berries	1/2 cup	
rosemary	1/2 tsp	
turmeric	1/2 tsp	
garlic	2 cloves	
green tea	2 cups	
oolong tea	3 cups	
Lean Meats	6 oz	daily
Low glycemic fruit	2 servings	daily
**Study Restricted Foods**		
Dairy	N/A	never
Grains	N/A	never
Legumes	N/A	never
Alcohol	N/A	never

**Table 3:** Differences in consumption of dietary components between groups.

Dietary components	Group	M	SD	T	P
Colorful Vegetables	Intervention	125.028	60.163	-3.310	0.002
	Control	64.225	53.092		
Cruciferous Vegetables	Intervention	65.722	31.906	-4.943	<0.001
	Control	22.125	22.027		
Greens	Intervention	40.417	20.529	-4.752	<0.001
	Control	10.475	18.323		
Fruits	Intervention	93.528	50.530	-1.634	0.111
	Control	67.700	46.885		
Beets	Intervention	21.917	9.153	-8.282	<0.001
	Control	1.850	4.931		
Pumpkin Seeds	Intervention	18.944	11.602	-6.291	<0.001
	Control	1.150	3.229		
Sunflower Seeds	Intervention	21.33	10.152	-8.112	<0.001
	Control	1.400	2.501		
Methyl Adaptogens	Intervention	59.611	35.139	-3.399	0.002
	Control	25.650	25.001		
Lean Meats	Intervention	41.333	28.493	-2.048	0.048
	Control	27.200	11.337		
Eggs	Intervention	26.111	4.626	-4.710	<0.001
	Control	15.250	9.088		
Liver	Intervention	9.194	5.342	-7.303	<0.001
	Control	0.000	0.000		
Methyltetrahydrofolate Change (nmol/L)	Intervention	8.056	16.444	-2.536	0.016
	Control	-3.850	12.393		
Grains	Intervention	9.083	12.764	2.125	0.041
	Control	18.750	15.028		
Legumes	Intervention	0.694	1.363	2.946	0.008
	Control	4.975	6.338		
Alcohol	Intervention	9.361	11.804	0.433	0.668
	Control	11.200	14.132		
Dairy	Intervention	4.250	8.198	4.466	<0.001
	Control	22.300	15.874		

The mean frequency of recommended and restricted foods consumed per month in the control and treatment groups. Dietary variables are organized according to study recommendations, from most to least consumed. The intervention group is represented by the blue bars, and the control group is represented by the green bars. The last four dietary variables are food groups that participants in the treatment group were told to avoid, including grains, legumes, alcohol, and dairy.

### Group differences in baseline epigenetic age, weight changes, and consumption of dietary variables

Significant differences in mean weight change between visit one and visit three (*p* = 0.009), baseline Epigenetic Age Acceleration (i.e., the difference between chronological age and epigenetic age) (*p* = <0.001), and consumption of most dietary variables (excluding alcohol and fruits) were found between the intervention and the control groups (Table 3). The mean change in blood levels of methyltetrahydrofolate, a proxy measure of adherence, was significantly increased in the intervention group, suggesting that they consumed more folate-containing foods such as cruciferous vegetables, dark leafy greens, and liver (Table 3). One participant in the intervention group was missing visit three MF; For this participant, MF levels measured on visit two were used to calculate change in MF. On average, those in the treatment group had an epigenetic age of 5.26 (*SD* = 4.96) years younger than their chronological age at baseline. In contrast, those in the control group were 0.816 (*SD* = 5.59) years older than their chronological age. The intervention group lost an average of 4.61 (*SD* = 7.91) pounds between the first and third study visits, while the control group gained an average of 0.90 (*SD* = 3.892) pounds.

Spearman’s Correlation Coefficients for Change in Epigenetic Age (EA), Baseline Epigenetic Age Acceleration (EAA), Change in Weight, and Recommended Foods before adjusting for weight change. Results are color-coded; positive correlations are yellow, and negative correlations are blue, with shades of green indicating the strength and direction of the correlation coefficient. The intensity of the color represents the strength of the correlation. For example, dark blue to blue-green indicates a strong negative correlation, while bright green to yellow indicates a positive correlation. Asterisks beside the variable name indicate significant correlations with changes in epigenetic age (*p* < 0.05).

Spearman’s Correlation Coefficients for Change in Epigenetic Age (EA), Baseline Epigenetic Age Acceleration, Change in Weight, and Restricted Foods are color-coded; Positive correlations are shown in yellow, and negative correlations are shown in blue, with shades of green indicating the strength and direction of the correlation coefficient. For example, dark blue to blue-green indicates a strong negative correlation, while bright green to yellow indicates a positive correlation. Asterisks beside the variable name indicate significant correlations with changes in epigenetic age (*p* < 0.05).

### Associations between changes in epigenetic age changes, dietary variables, weight changes, and baseline epigenetic age acceleration (ρ)

**Figure 2:**
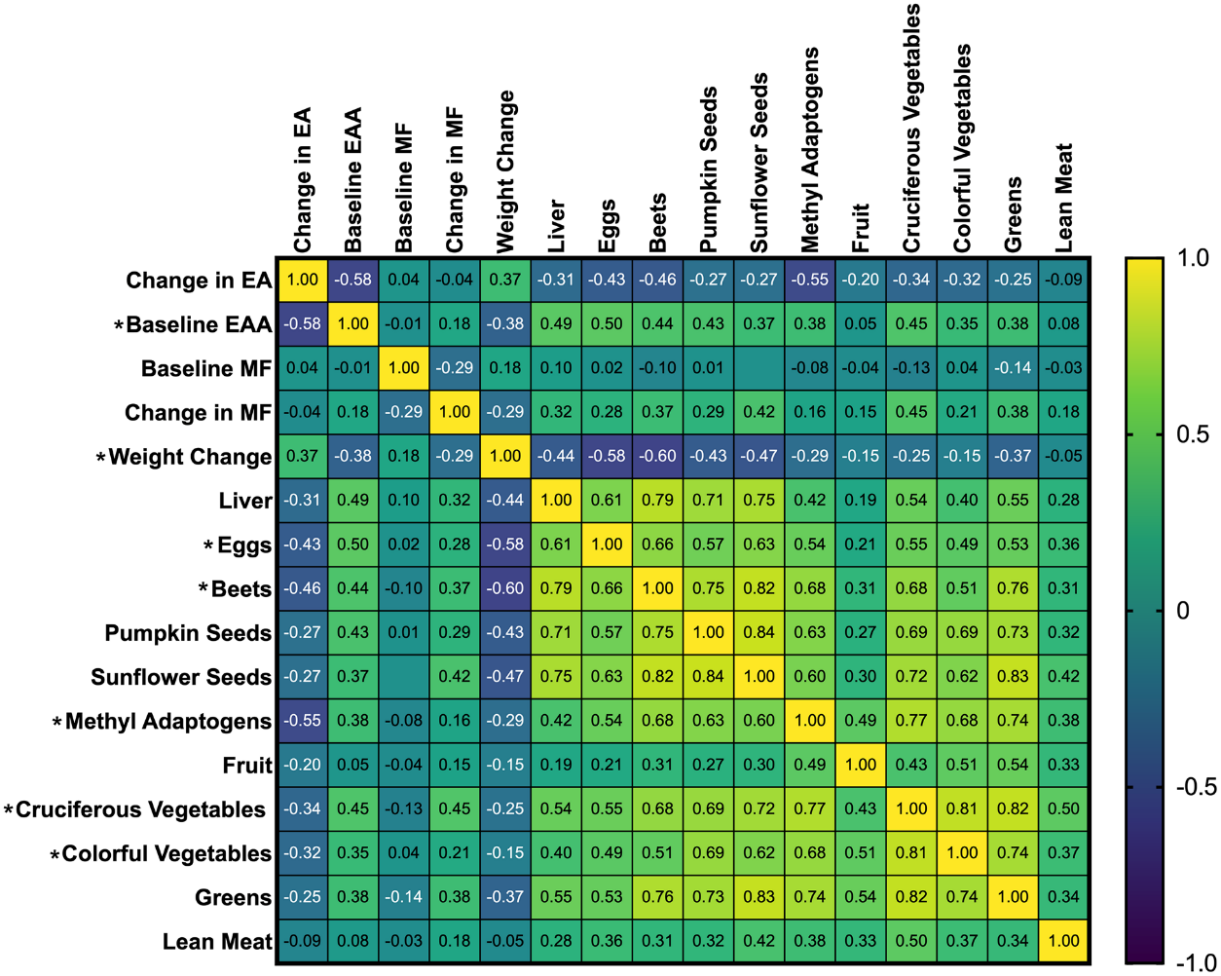
Spearman’s correlation coefficients of study recommended foods, baseline EAA, and change in epigenetic age.

**Figure 3:**
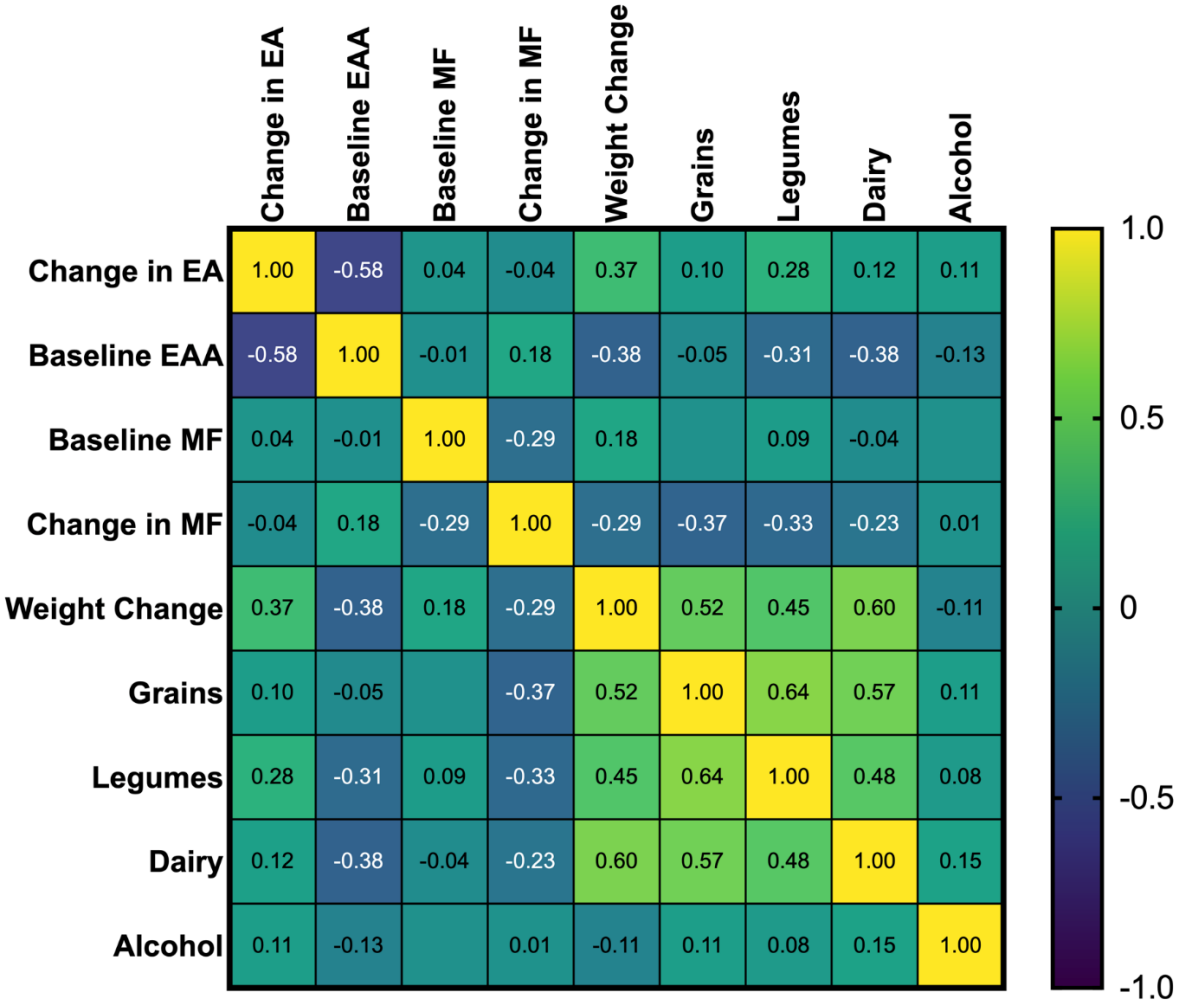
Spearman’s correlation coefficients of study restricted food groups and baseline EAA, and change in epigenetic age.

Significant unadjusted negative correlations were observed between changes in epigenetic age and baseline epigenetic age (*r_s_ = -*0.58, *p* = <0.001), eggs (*r_s_ =* -0.43*, p* = .007), beets (*r_s_ =* -0.46*, p* = 0.004), methyl adaptogens (*r_s_ =* -0.55, *p* = <0.001), cruciferous vegetables (*r_s_ =* -0.34*, p* = .034), and colorful vegetables (*r_s_ =* -0.32*, p* = 0 .047) (Figure 1). Weight change was positively correlated with epigenetic age change (*r_s_ =* 0.37*, p* = 0.021) (Figure 2). In general, dietary variables were highly correlated with each other. None of the restricted foods were significantly correlated with epigenetic age change, although there was a trend towards increased consumption of those foods and increased epigenetic age (Figure 3). However, neither group consumed any restricted foods once per day or more on average (Figure 1). After adjusting for weight change, only baseline EAA (*r_s_ =* -0.507*, p* = <0.001) and the methyl adaptogens (*r_s_ =* -0.498*, p* = 0.002) remained significantly negatively correlated with epigenetic age change and were chosen to be further analyzed along with weight change in a Hierarchical linear regression model (Table 4).

**Table 4:** Adjusted correlations between frequency of dietary intake and changes in epigenetic age.

**Partial spearman’s correlations**		
**Adjustment variable: weight change**	**Change in EA**	***p-*Values**
Baseline EAA	**-0.507**	**0.001**
Liver	-0.177	0.294
Eggs	-0.281	0.092
Beets	-0.317	0.056
Pumpkin Seeds	-0.13	0.441
Sunflower Seeds	-0.119	0.483
Methyl Adaptogens	**-0.498**	**0.002**
Cruciferous Vegetables	-0.279	0.095
Colorful Vegetables	-0.292	0.079
Greens	-0.131	0.441
Fruit	-0.151	0.372
Lean Meat	-0.078	0.648

The partial Spearman’s correlation coefficients are shown above and represent the relationship between change in EA, baseline EAA, and study-specific dietary recommendations with weight change as an adjustment variable. Significant *p*-values are bolded.

### Linear regression analysis

In Model 1, a significant linear association was found between *ln*(methyl adaptogens) and epigenetic age change (*p* < 0.001), with higher consumption predicting a reduction in epigenetic age. The relationship remained highly significant after adjusting for weight change in Model 2 (*p* = 0.001). When baseline EAA was included as an adjustment variable in Model 3, with weight change, the relationship between epigenetic age change and methyl adaptogens remained significant, but the strength of the association was attenuated (Table 5). Baseline EAA was significantly associated with epigenetic age change (*p* = 0.007), with higher values of baseline EAA predicting a reduction in epigenetic age.

**Table 5:** Adjusted associations between methyl adaptogens and change in epigenetic age.

**Independent variables**	**Unstandardized *beta***	***95% CI***	***p***	***R***	***R^2^***	***DeltaR^2^***	***F*-change**	**Sig. *F***
**Model 1**				0.541	0.292	0.292	14.864	**<0.001**
*ln*(Methyl Adaptogens)	-1.828	-2.8, -.87	**<0.001**					
**Model 2**				0.554	0.307	0.015	0.767	0.387
*ln*(Methyl Adaptogens)	-1.729		**0.001**					
Weight Change	0.082	-.11, .27	0.387					
**Model 3**				0.665	0.442	0.134	8.189	**0.007**
*ln*(Methyl Adaptogens)	-1.214		**0.016**					
Weight Change	0.048		0.577					
Baseline EAA	-0.288	- 49, -.08	**0.007**					

The results of the hierarchical linear regression analysis are shown above. The methyl adaptogen variable was natural log-transformed (*ln)* to meet normality assumptions. Weight change was calculated by subtracting the difference between weight at visit 3 from baseline weight. Baseline EAA is measured as the difference between chronological and epigenetic ages at visit 1. Model 1 includes *methyl adaptogens:* Model 2: Model 1 + *weight change*; Model 3: Model 2 + *baseline EAA*. Significant *p*-values are bolded.

## DISCUSSION

This secondary analysis of the Methylation Diet and Lifestyle trial aimed to identify associations between specific features of the study diet (Table 2) and non-dietary factors potentially influencing changes in epigenetic age (Figures 2, 3 and Tables 4, 5). This was done to understand better the observed variability in epigenetic age changes across groups. Results from the regression analysis suggest that higher consumption of methyl adaptogens, a category of foods chosen for their potential to be polyphenolic modulators of DNA methylation (turmeric, garlic, berries, green tea, oolong tea, and rosemary), was strongly associated with reductions in epigenetic age after adjusting for weight changes and baseline epigenetic age acceleration (*p* = 0.016) (Table 5). These findings align with a growing body of research elucidating the protective role of polyphenol consumption in biological aging [21–25].

Polyphenols in methyl adaptogens may reduce epigenetic aging through multiple mechanisms. One such pathway involves the modulation of DNA methyltransferases, enzymes responsible for adding methyl groups to DNA. Epigallocatechin gallate (EGCG) from green tea, allicin from garlic, anthocyanins from berries, curcumin from turmeric, and rosmarinic acid from rosemary have been shown to inhibit DNA methyltransferases and regulate the expression of genes associated with accelerated epigenetic aging [26–30]. For example, EGCG, allicin, anthocyanins, and rosmarinic acid modulate the PIK3/AKT/mTOR signaling pathways mediated by the PIK3CB gene. These pathways govern cellular functions, including proliferation, metabolism, and survival, and disruptions are implicated in aging-related diseases such as cardiovascular, endocrine, and neurodegenerative conditions, as well as cancer [31–36].

Several foods recommended in the methyl adaptogens group also contain compounds that influence telomerase via gene expression [37–40]. Telomeres are protective, repetitive DNA sequences responsible for maintaining chromosomal integrity; progressive shortening of these complexes eventually leads to cell death [41]. Although telomere shortening has been implicated in accelerated aging, high cellular levels of telomerase are a hallmark of cancerous cells [42]. Preclinical studies indicate that EGCG, curcumin, allicin, anthocyanins, and rosmarinic acid selectively induce apoptosis in malignant cells while maintaining telomere integrity in healthy cells, emphasizing the potential of compounds found in methyl adaptogens to safely mitigate aging processes [38, 43, 44]. However, human evidence is limited, and long-term studies are needed.

Related to our research, recent studies continue to evaluate the role of polyphenols consumed as part of a healthful dietary pattern in epigenetic aging. For example, Kawamura et al. found that Japanese men who consume a traditional Japanese diet rich in vegetables, green tea, and seafood exhibited lower epigenetic age than those following a Western diet [22]. Similarly, observational and interventional studies have identified associations between polyphenol-rich Mediterranean diets and epigenetic age attenuation. A cross-sectional study of 5,000 individuals in Italy revealed that adherence to a polyphenol-rich Mediterranean diet was linked to lower epigenetic age and that this effect was beyond the antioxidant activity of these foods [25]. Furthermore, a one-year pilot study of a Mediterranean dietary intervention demonstrated a trend toward epigenetic age reversal, which became significant among participants who were epigenetically older at baseline. Consistent with these findings, our analysis also observed a significant relationship between baseline Epigenetic Age Acceleration (EAA) and changes in epigenetic age. However, higher consumption of methyl adaptogens remained significantly associated with epigenetic age reduction even after adjusting for baseline EAA.

The DIRECT PLUS study measured extrinsic and intrinsic epigenetic age in participants following a green Mediterranean dietary intervention rich in vegetables, walnuts, green tea, and makai. Researchers discovered an association between certain dietary polyphenols, polyphenol metabolites, and slower biological aging, specifically tyrosols and urolithins [21, 45]. Tyrosols and hydroxytyrosols are phenolic compounds found in olive products and, to a lesser extent, green tea [46]. Urolithins are microbial metabolites of a grouping of polyphenols known as ellagitannins found in pomegranates, walnuts, seeds, and some berries [47, 48]. Tyrosols and urolithins have been explored for their potential anti-aging, anti-inflammatory, and anticarcinogenic properties [49–53]. Combining these polyphenols, polyphenol metabolites, and others in methyl adaptogen foods could potentiate beneficial effects. For example, one study investigating EGCG and urolithin A in a mouse model of late Alzheimer’s disease found that combining the two was more effective at stimulating mitophagy, lessening mitochondrial dysfunction, and improving coordination, learning, and memory than urolithin A supplementation alone. This suggests that consuming bioactive compounds from polyphenols from varied sources, like the foods in the methyl adaptogen grouping, may enhance beneficial effects [54].

Interestingly, weight changes throughout the study did not significantly predict reductions in epigenetic age in the regression model, differing from research that links caloric restriction to delayed biological aging (Table 5) [55]. Similarly to our study, Horvath et al. found that a short-term reduction in BMI was not associated with epigenetic age reversal [56]. In contrast, longer-term studies have found an association between weight loss and epigenetic age attenuation [57, 58]. The effects of methyl adaptogens may operate independently of caloric intake, possibly through direct modulation of epigenetic regulators. Additionally, the effects of weight loss on epigenetic markers of aging could require longer-term data to be accurately quantified.

### Variability of change in epigenetic age

Although fundamentally an association analysis, the fully adjusted regressions model developed in these analyses accounted for 44% of the variability observed in epigenetic age change during the parent clinical trial. Previous research has suggested that up to 40% of the variability in Horvath’s algorithm is genetically determined. Together, these changes may account for significant amounts of observed variability. Although genetic differences may be directed to differences between epigenetic age and chronological age at baseline, these findings may not be independent [59, 60].

### Strengths and limitations

The most salient challenge in this analysis was the sample size (n = 38), resulting in insufficient power to include more covariates in the linear regression analysis. Including all participants as a cohort introduced a significant skew in the dietary variables. Additionally, collinearity was observed between dietary variables, and as such, another limitation is the inability to assess some dietary recommendations independently of each other. The methyl adaptogen variable was log-transformed to meet assumptions of normality, which could influence the strength of the association with change in epigenetic age and make interpretation less intuitive.

A common constraint in clinical trials investigating a dietary intervention is reliance on self-reported dietary data, as participant bias could influence the strength of the associations. However, VioScreen and a study-specific questionnaire were employed to capture dietary intake accurately. Additionally, blood levels of methyltetrahydrofolate were significantly higher in the intervention group at the end of the study, indicating the participants were eating study-recommended foods rich in folate, such as broccoli, dark leafy greens, cabbage, liver, and berries (Table 3) [61, 62].

Other components of the lifestyle intervention were not included in this analysis but may have contributed to the variability in measures of epigenetic age. Exercise was very high in the intervention and control groups; therefore, activity changes during the trial are less likely to have influenced changes. However, sleep and adherence to the recommendation to practice stress reduction in a defined relaxation response meditation were not tracked and cannot be assessed. Smoking was not included in the analysis as none of the men enrolled were active smokers, but smoking history could have influenced epigenetic age at baseline. Gao et al. found that self-reported smoking history was not associated with accelerated epigenetic aging as measured by the Horvath and Hannum models, despite confirming that accelerated epigenetic age was associated with smoking-related CpGs [63]. Despite the limited sample size and adjustment variables, the methyl adaptogen variable retained significance, and the overall model accounted for nearly half of the variability in epigenetic age change, a strength in this study, considering the potential environmental and genetic factors that influence epigenetic age.

Lastly, the participants were all male and mostly white (81%). This is both a strength and a limitation in this analysis. The relative homogeneity of the group reduced potential confounders but decreased the generalizability of the findings to a broader population. However, a recent case series was published purporting to replicate the original study’s results in a group of six women [64]. Although the results support the MDL study findings, conclusions cannot be drawn from an uncontrolled case series of six women.

Horvath’s 2013 pan-tissue algorithm was used to measure epigenetic age in the original study as this measure is reliably robust when evaluating multiple tissue types, including saliva. As genetic samples were derived from saliva in this cohort, evaluating epigenetic age via well-known second and third-generation algorithms trained using other tissue types (e.g., blood) could lead to unreliable results [65, 66]. Therefore, our analysis is restricted to the original EA outcome measure. Each epigenetic aging algorithm contains unique CpGs related to the aging process. We recognize that utilizing multiple algorithms could allow for a better understanding and assessment of the clinical relevance of the findings, and therefore, restricting our analysis is a limitation of this study. This research was an exploratory secondary analysis; results are intended to be hypothesis-generating by exploring dietary factors related to the intervention associated with epigenetic age changes.

### Conclusions and future directions

The results of this secondary analysis of the Methylation Diet and Lifestyle trial suggest that consumption of foods categorized as methyl adaptogens due to their potential to be polyphenolic regulators of DNA methylation, including turmeric, green tea, oolong tea, rosemary, garlic, and berries, were associated with epigenetic age reduction in healthy, middle-aged men. Participants epigenetically older than their chronological age at baseline were more likely to exhibit a reversal in epigenetic age. Changes in weight throughout the study, potentially due to unintentional caloric restriction, did not predict epigenetic age reversal. Future research should include a more extensive and diverse population to enhance generalizability and utilize extrinsic epigenetic age measures to determine the intervention’s relevance in mitigating future morbidity and mortality, as extrinsic epigenetic age has been shown to predict adverse health outcomes more accurately in epidemiological data [67].

## MATERIALS AND METHODS

### Study design and conduct

This study is a secondary data analysis of de-identified data from the Methylation Diet and Lifestyle study, a randomized controlled trial in healthy adult men (n=43) between 50-72 that tested epigenetic age before and after a diet and lifestyle intervention and applied Horvath’s algorithm as a primary outcome measure [13]. The original trial was approved by the Institutional Review Board of the National University of Natural Medicine (IRB: RB100217) and was registered on ClinicalTrials.gov (NCT03472820). All participants provided written consent before enrollment, including consent for data to be used for future research. The study strictly adhered to ethical principles outlined by the Declaration of Helsinki. The intervention consisted of a specialized diet designed to modulate DNA methylation, thirty minutes of moderate to vigorous exercise five days per week, seven hours of sleep nightly, and twenty minutes of daily meditation. Data were collected during three study visits: baseline, week five, and week nine. Dietary data recorded during week nine were used for this analysis as they most accurately represent participants’ consumption of study-specific foods.

### Participants

The study participants were 43 healthy adult men aged 50-72 recruited from Portland, Oregon, USA without a recent or chronic disease history. This age group was chosen as it is a time period when age-related changes begin to manifest. For rationale regarding excluding women and other age groups, refer to the methods section of the original study at https://pubmed.ncbi.nlm.nih.gov/33844651/. See Table 1 for details of participant demographics. Thirty-eight men completed the trial. Data missing from the five participants were not included in this analysis.

### Dietary guidelines

The dietary guidelines incorporated daily consumption of dark leafy greens, cruciferous vegetables, colorful vegetables, pumpkin or sunflower seeds, beets, lean meats, low glycemic fruit, and a serving or more from a group of foods categorized as ‘methyl adaptogens,’ i.e., foods containing vitamins or polyphenolic components shown in mechanistic studies to modulate the methylome [12–16]. Participants could choose one or more items from the methyl adaptogen group daily, including turmeric, rosemary, garlic, green tea, or oolong tea. Liver was recommended three times per week. See Table 2 for details. Although research on whole grains, legumes, and certain dairy products supports the consumption of these foods as health-promoting, [68, 69] they were omitted from the study diet to reduce the potential for short-term gastrointestinal side effects [70–72].

### Genetic sampling and DNA methylation analysis

DNA methylation was measured and analyzed at the Yale Center for Genome Analysis [13]. In brief, genetic information was derived from saliva samples and DNA normalized to 1 μg, for the Zymo EZ-96 DNA Methylation Kit (Cat. No. D5004) before undergoing an overnight bisulfate conversion and purified using the Zymo Methylation protocol. DNA was analyzed using Illumina Methylation Epic Arrays (Cat. No. WG-317- 1001). Horvath’s clock, available online at https://dnamage.genetics.ucla.edu/, was used to measure epigenetic age at baseline and post-intervention. For more details on the DNA methylation analysis, see the methods section of the original study at https://pubmed.ncbi.nlm.nih.gov/33844651/.

### Outcome variable

The outcome variable in this study is the change in age per Horvath’s clock (2013) as a measure of epigenetic age, calculated as the difference between epigenetic age measured at week nine and subtracted by baseline epigenetic age.

### Dietary variables

Participants self-reported consumption of study-specific foods during three study visits using the VioScreen (by VioCare, Princeton, NJ, USA) food frequency questionnaire (FFQ). Questions were answered on a standard FFQ scale from 1-8 (1 = never, 2 = 1x per month, 3 = 2-3x per month, 4 = 1x per week, 5 = 2x per week, 6 = 3-4x per week, 7 = 5-6x per week, 8 = every day). Dietary variables were constructed using the FFQ data and categorized according to study guidelines. Original responses recorded on a scale were transformed into continuous variables representing the estimated times a food was consumed monthly. For example, if a participant reported their consumption of a particular food as a “5” on the FFQ scale (5 = 2x per week), their frequency of consumption per month for that food was recorded as “8x per month”. For cases where an individual answered the FFQ with a number representing a range of consumption (e.g., 7 = 5-6x per week), the average was taken to produce an estimated frequency of consumption per month (7 = 22x per month).

For recommendations encompassing categories of foods (e.g., cruciferous vegetables), the consumption of the recorded foods in that category was summed to represent the total number of times that food group was consumed monthly. In total, eleven variables represented study dietary recommendations, and four variables represented study-restricted foods. Variables in the recommended category included eggs, beets, liver, colorful vegetables, cruciferous vegetables, dark leafy greens, low glycemic fruit, lean animal meats, pumpkin seeds, sunflower seeds, and methyl adaptogens. Variables representing restricted foods were legumes, dairy, alcohol, and grains. Participants were also asked to avoid added sugars and trans fats; however, the FFQ questionnaire did not capture sugar intake, and participants did not report consuming trans fats.

Baseline blood levels methyltetrahydrofolate (MF) and changes MF levels measured in nanomoles per liter (nmol/L) will be used as a proxy measure of participant consumption of folate-rich foods throughout the study. Baseline levels of MF and MF change will be explored for association with baseline epigenetic age acceleration and epigenetic age changes, as folate influences the epigenome through one-carbon metabolism. The most recent MF measurements available (e.g., from study visit 2) will be used to calculate MF change in the case of missing data.

### Adjustment variables

Variables with the potential to influence the variability of epigenetic age change include weight change during the trial and the difference in epigenetic age and chronological age at baseline, which we will refer to as baseline Epigenetic Age Acceleration (EAA). Horvath termed Epigenetic Age Acceleration to describe the difference between epigenetic and chronological age [8]. Participant demographics were not considered as the group was fairly homogenous; participants were mostly white men (81.9%), between the ages of 50-72, and highly educated, with more than half reporting completing a graduate degree in the treatment group.

### Statistical analysis

In this exploratory secondary data analysis, group allocation was omitted, as the study’s goal is to explore predictive factors underlying the variability in epigenetic age change across both the treatment and control groups, emphasizing study-specific dietary recommendations. Normality was assessed via histograms and the Shapiro-Wilk test of normality. As expected, there were between-group differences in the frequency of study-specific food consumption, and dietary variables were abnormally distributed.

Two-sided independent sample T-tests were performed to determine if there were significant between-group differences in mean weight change between visits one and three and baseline differences between epigenetic age and chronological age (baseline EAA), as well as dietary variables between the intervention and control groups.

Spearman’s rank correlation was used to examine correlations between epigenetic age change, baseline epigenetic age acceleration (EAA), weight change, and use of recommended and restricted foods as a robust statistical test known for its ability to analyze non-parametric data and resilience against outliers in small data sets [73, 74]. After the initial correlation analysis, partial Spearman’s correlations were utilized to adjust for weight change on the effects of the dietary variables and baseline EAA on epigenetic age change to identify dietary variables for further analysis.

After adjusting for weight change, variables that remained significantly correlated were chosen for further analysis using hierarchical linear regression, with change in epigenetic age maintained as the dependent variable. Dietary variables were entered first to determine their unique impact on variability in epigenetic age before adjustment. Abnormally distributed variables were log-transformed. All analyses were performed using SPSS Version 29 (IBM Corp., Armonk, NY, USA), [75], and graphs and correlation matrices were generated using GraphPad Prism (version 10).
